# Realizing the promise of machine learning in precision oncology: expert perspectives on opportunities and challenges

**DOI:** 10.1186/s12885-025-13621-2

**Published:** 2025-02-17

**Authors:** Vasileios Nittas, Kelly E. Ormond, Effy Vayena, Alessandro Blasimme

**Affiliations:** 1 Epidemiology, Biostatistics and Prevention Institute, University of Zurich, Hirschengraben 84, Zurich, 8001 Switzerland; 2 Health Ethics and Policy Lab, Department of Health Sciences and Technology, Swiss Federal Institute of Technology (ETH Zurich), Hottingerstrasse 10, Zurich, 8092 Switzerland

**Keywords:** Precision oncology, Artificial intelligence, Machine learning, Ethics, Algorithmic bias, Bias, Explainability, Opacity, Black box, Privacy

## Abstract

**Background:**

The ability of machine learning (ML) to process and learn from large quantities of heterogeneous patient data is gaining attention in the precision oncology community. Some remarkable developments have taken place in the domain of image classification tasks in areas such as digital pathology and diagnostic radiology. The application of ML approaches to the analysis of DNA data, including tumor-derived genomic profiles, microRNAs, and cancer epigenetic signatures, while relatively more recent, has demonstrated some utility in identifying driver variants and molecular signatures with possible prognostic and therapeutic applications.

**Methods:**

We conducted semi-structured interviews with academic and clinical experts to capture the status quo, challenges, opportunities, ethical implications, and future directions.

**Results:**

Our participants agreed that machine learning in precision oncology is in infant stages, with clinical integration still rare. Overall, participants equated ongoing developments with better clinical workflows and improved treatment decisions for more cancer patients. They underscored the ability of machine learning to tackle the dynamic nature of cancer, break down the complexity of molecular data, and support decision-making. Our participants emphasized obstacles related to molecular data access, clinical utility, and guidelines. The availability of reliable and well-curated data to train and validate machine learning algorithms and integrate multiple data sources were described as constraints yet necessary for future clinical implementation. Frequently mentioned ethical challenges included privacy risks, equity, explainability, trust, and incidental findings, with privacy being the most polarizing. While participants recognized the issue of hype surrounding machine learning in precision oncology, they agreed that, in an assistive role, it represents the future of precision oncology.

**Conclusions:**

Given the unique nature of medical AI, our findings highlight the field’s potential and remaining challenges. ML will continue to advance cancer research and provide opportunities for patient-centric, personalized, and efficient precision oncology. Yet, the field must move beyond hype and toward concrete efforts to overcome key obstacles, such as ensuring access to molecular data, establishing clinical utility, developing guidelines and regulations, and meaningfully addressing ethical challenges.

**Supplementary Information:**

The online version contains supplementary material available at 10.1186/s12885-025-13621-2.

## Background

The ability of machine learning (ML) to process and learn from large quantities of heterogeneous patient data is gaining attention in the precision oncology community [1–6]. Some remarkable developments have taken place in the domain of image classification tasks in areas such as digital pathology and diagnostic radiology.

The application of ML approaches to the analysis of DNA data, including tumor-derived genomic profiles, microRNAs, and cancer epigenetic signatures, while relatively more recent, has demonstrated some utility in identifying driver variants and molecular signatures with possible prognostic and therapeutic applications [7, 8].

High-throughput sequencing enables fast and reliable mapping of germline and somatic DNA variants and high-resolution transcriptomic and proteomic analyses, which, combined with other emerging methods such as liquid biopsies, are expected to significantly advance the field [9–11]. ML’s success in other areas offers hope for similar advances in precision oncology, including the otherwise cumbersome analysis of high-throughput data [5].

Such advances span a broad array of applications, from drug discovery to diagnosis, risk prediction, and treatment decision-making, including the choice of patient-specific pharmacological regimens. Current evidence is positive, with ML algorithms often outperforming standard methods, such as identifying cancers with DNA repair defects and predicting radiation response [12, 13]. The growing role of ML has been driven by initiatives such as the Cancer Genome Atlas (TCGA), which has enabled the use of genomic data for the development and training of ML algorithms [14]. Since its pilot in 2006, TCGA has created enormous value for cancer research, generating evidence on genomic and molecular changes across various cancer types [15]. Recent advances in integrating multi-omic data (e.g., DNA and RNA data, epigenetic signatures such as methylation state, and gene expression profiles) also show considerable prospects for further development. Applications include cancer subtyping, patient stratification, cancer progression, and clinical outcome prediction.

Despite the growing role of ML in cancer research, its clinical implementation is still in its infancy [6]. Moving beyond proof-of-concept stages to real clinical impact is still far from reality and requires a better understanding of the hurdles ahead [5].

## Methods

The use of AI in precision oncology is a novel and evolving topic. For this reason, we decided to conduct semi-structured qualitative interviews with academic and clinical experts in precision oncology and ML (or AI). The ETH Zurich Ethics Commission granted approval. This methodology aims not to offer a representative picture of professionals’ attitudes but to start charting opportunities and challenges in this space. This study explores the challenges and opportunities linked to the clinical translation of ML-driven precision oncology. Through the views of academic and clinical experts, we identify and discuss the field’s current state and its perceived strengths, obstacles, and associated ethical challenges. Our work is among the first to systematically assess ML prospects in precision oncology. Thus, it fills an essential gap in the literature, as little attention has been paid to the impact of ML and artificial intelligence (AI) in this field.

### Sampling and recruitment

Participants were identified and recruited by convenience sampling. A list of potential interviewees was compiled from contacts of the research team and collaborators, without geographic limitation, followed by snowball sampling from those who replied to our invitation. Each invitation included an information sheet briefly outlining the study aims and participant rights. Reminders were sent two to three weeks after initial contact. Those who responded positively were scheduled for video-conference interviews and were sent the informed consent sheet to digitally sign and return before the interview. Interviews were concluded when thematic saturation was reached, meaning no significant new information was being contributed.

### Interview process and data collection

Interviews were guided by a semi-structured guide developed by a research team of two bioethicists (EV, AB), one genetic counselor with clinical ethics training (KO), and one epidemiologist (VN), as well as an external expert (oncologist). It consisted of three parts: first, questions about the participants’ background and experience; second, questions on precision oncology and the field’s current state; and third, questions about ML use in precision oncology. The first two interviews were used to test the interview guide’s flow and gaps and were followed by minor adjustments. Interviews were conducted by a single interviewer (VN) via Zoom between March and June 2022. Verbal permission to record the session (audiovisual) was sought at the beginning of each interview.

Interviews were transcribed within two days of completion using the automatic transcription software Otter.ai. Transcriptions were quality checked by VN, corrected manually, and deidentified by deleting any references that could reveal the interviewee’s identity (e.g., names, specific projects, work department), with each interview assigned to a participant ID. The recordings were deleted shortly after transcription.

### Analysis and synthesis

The analysis was based on an iterative, guided thematic framework analysis method [16]. A deductive codebook was developed a-priori, based on the interview guide and expected topics. Our analytical approach was divided into four phases. First, we familiarized ourselves with the data by reading the interviews. During that process, we expanded and internally discussed the codebook, with topics emerging from the data (inductive coding). Second, all interviews were coded by VN using the MAXQDA software. Third, codes were compared. Fourth, similar or related codes were combined into overarching themes. Themes captured the essence of related codes in one single topic. Results were synthesized narratively according to these themes. Below, we present themes mentioned by over half the participants, as well as outliers that seemed critical.

## Results

We invited 97 experts; 88 replied, and 17 participated. Five were female. Most worked in Europe (*n* = 12), and five in the US. At the time of the interviews, they had roles in academic research (*n* = 13), private sector (*n* = 2), government (*n* = 1), and clinical settings (*n* = 1). Thirteen used AI/ML in their work, primarily for diagnostics and predictions. Participants self-assessed their ML knowledge as intermediate (*n* = 7) or advanced (*n* = 10). Table 1 provides an overview of participant characteristics.

**Table 1:** Participant characteristics

Gender	Occupation	Main area of expertise	Geographic location
Male	Professor	Oncology	Italy
Female	Scientist	Precision medicine	France
Male	Physician	Precision oncology	USA
Female	Professor	Molecular Genetics	USA
Male	Professor	Genomics	Switzerland
Male	Scientist	Molecular Biophysics	Sweden
Female	Scientist	Oncology	Germany
Male	Professor	Bioinformatics	Cyprus
Male	Professor	Biology	France
Male	Biologist	Molecular oncology	France
Male	Physician	Oncology	France
Male	Professor	Biomedical informatics	USA
Male	Scientist	Oncology	France
Female	Professor	Medical ethics	USA
Male	Scientist	Oncology	Italy
Male	Professor	Bioengineering	USA
Female	Scientist	Oncology	Germany

### Status quo

When asked about the status quo, participants emphasized that precision oncology is still far from standard care. They underlined that genomic sequencing currently plays a limited clinical role, primarily in large, specialized centers. Reasons for this included insufficient clinical validation and a lack of financial and infrastructural resources, including transparent reimbursement schemes. It was also noted that success stories of precision oncology have, until now, been scarce.

“*Right now the success stories are for the tumors which are quite homogeneous and driven by one molecular alteration.” [INT 11]*

Some interviewees described knowledge gaps. Although approved targeted therapies exist, there are missing linkages between drugs and genetic alterations. Others highlighted challenges in access, with only a small percentage of cancer patients currently benefiting from targeted therapies due to lack of insurance coverage or not fully meeting requirements. Participants suggested that the field should start targeting cancer types with multiple and rarer molecular alterations to widen clinical benefits.

“*I think at present time*,* the major bottleneck is really in the in the development of better drugs*,* and better connections between the treatment and the genetic alteration.” [INT 14]*

When explicitly asked about the status quo of ML in precision oncology, participants emphasized that it is in very infant stages. They stressed that the application of ML is mostly limited to research and the identification of biomarkers, not clinical ones. One participant argued that one of the main reasons for this is the lack of motivation among ML researchers to go all the way toward clinical implementation. Despite this, most participants were enthusiastic and aware of ML’s potential contribution to the future of cancer care, as outlined in the next section.

*“…and we actually went to find real projects that were doing machine learning in precision medicine*,* and we couldn’t find any…” [INT 16]*

### Perceived strengths

When asked about the specific strengths of ML, all participants were able to mention at least one. Participants equated ML use with improved and faster treatment decisions and treatment response predictions. They emphasized the added value of ML as being able to analyze data from many tumors to better identify which patients, even those with multiple molecular alterations, will not benefit from existing targeted therapies. They also underlined the ability of ML to deal with the dynamic nature of cancer and break down the high complexity of molecular data.

*“…being able to say […] with this array of changes in this proportion of the tumor cells*,* this is what’s most likely to work*,* it’s going to take things like AI and machine learning. Yeah*,* individual pathologists aren’t going to be able to do that.” [INT 2]*

*“…you can definitely improve the care of patients based on those molecular data*,* but to really get into molecular data and interpret and get the best of the patterns in the data*,* you have to have machine learning.” [INT 11]*

Beyond that, participants mentioned the organizational impact of ML. Simpler ML models could support clinicians with routine clinical tasks, such as identifying the right treatments faster and matching patients to available clinical trials, reducing the burden for molecular tumor boards, and improving clinical workflows. The clinical role of ML was often described as assistive and supportive rather than prescriptive, including supporting the identification of a clinical trial that fits the patient’s circumstances.

*“…so another more organizational impact ML or AI could have is*,* as we just said*,* there are many targeted agents*,* many treatments*,* many trials with targeted agents*,* […] so a more automatized way […] to match a patient to a trial or compound could…” [INT 12]*

*“so you know*,* rather than having a horde of residents*,* you know*,* digging literature in search for the best trial for that patient*,* you have a computer coming up with a shortlist [INT 14]*

### Perceived obstacles

We asked participants what obstacles must be overcome for ML models to be clinically integrated. Their responses fell into three sub-themes: data access, sharing, and reuse; clinical utility; guidelines and regulation.

### Data access, sharing, and reuse

Access to and use of molecular data was a repeatedly mentioned obstacle. Data availability and access were described as essential to the advancement of ML in oncology and its future clinical implementation. Almost all participants emphasized the difficulty of accessing big, multidimensional, and structured molecular data for research purposes, especially in highly regulated settings such as the European Union. Centers that hold large volumes of molecular data and have the resources to handle these volumes are not well connected to each other or to other research facilities, making data-sharing and reuse challenging. Participants described that even when data are available, they may not be accessible or usable (e.g., because they are unstructured or insufficiently annotated).

*“This step is difficult*,* because it requires an amount of data that we are currently still struggling to get*,* especially because it’s difficult to really harvest this data [INT 14]”*

*“My theory is if you spend a tiny proportion of the money used to develop a new drug to collect the genetic data from the real patients*,* you will potentially have more benefit than any of these individual drugs” [INT 13]*

Participants emphasized that difficulty in accessing molecular data hinders ML development. To fully capture cancer’s complexity and to be clinically relevant, ML tools must be able to combine molecular data with other types of information, including clinical and contextual data (e.g., patient-generated data), as well as other -omic data. Regarding the current data ecosystem, participants described a vicious cycle, with the lack of data access hindering clinical utility, reducing motivation to facilitate access and faster clinical integration. Transfer learning, which allows algorithms to reuse learned patterns from related datasets, has been suggested as one solution to data scarcity.

*“…no Institute is trying to properly collect genomic data and clinical data together to enable training AI models” [INT 13]*

### Clinical utility

While participants held an overall positive attitude towards precision oncology, most agreed that its clinical utility is not established yet. Currently, most cancer patients do not benefit from genomic testing. The most often mentioned reason for that is the lack of evidence between diagnostics and targeted therapeutics. Targeted therapies do not yet exist for most cancers; if they exist, they are effective only short-term before the tumor develops resistance.

It was emphasized that clinical utility must first be verified for ML models to achieve clinical relevance. Until that point, the availability of genomic-based ML tools in clinical practice will add little value for most patients.

*“I think at present time*,* the major bottleneck is really in the in the development of better drugs*,* and better connections between the treatment and the genetic alteration […] We simply need to make a lot of hypotheses and test them […] And until we reach that stage*,* it’s probably relatively less important to have an AI-based tool that immediately identifies the best treatment…” [INT 14]*

Three participants described that it is difficult to distinguish those best suited for precision oncology tasks within the abundance of ML algorithms. Establishing clinical utility is further complicated by the heterogeneous nature of cancer, which likely reduces the overall performance of ML. For rare molecular alterations, patient subgroups become smaller and data scarcer. Participants also believed that robust evidence, held to the same standards as any other medical procedure, would likely increase trust and foster adoption.

### Guidelines and regulations

Responses regarding guidelines and regulations were divided between European and US participants. Although ML is not a magic bullet that should shortcut regulatory controls, it was repeatedly emphasized (primarily by participants from the European region) that the currently complicated legislative landscape (e.g., GDPR and its varying implementation across member states) may hinder data access and sharing. The lack of guidelines and agreed-upon standards further complicates this. This balance between regulation and data access was not a topic raised by participants from the US. Two participants underlined the need for regulatory frameworks that foster equitable and safe access to molecular data, complemented by education and well-defined ethical and practical guidelines.

*“What we need to do*,* at least in science*,* is to have a new regulatory framework in order to guarantee equitable access of all this data…” [INT 6]*

*“It’s not because it’s AI or ML*,* that it’s a magic bullet that should shortcut all the regulatory controls. But it’s not because it’s AI that we should say*,* oh*,* no*,* we need to wait another decade*,* before using it.” [INT 1]*

### Ethical challenges

Almost all participants agreed that several ethical challenges must be addressed before ML becomes routine in cancer care. Most frequently mentioned were privacy risks, equity, explainability and trust, and incidental findings.

### Privacy

Though quite mixed, opinions around privacy focused on handling molecular data used to develop, train, and validate ML algorithms. Five participants were highly concerned about privacy, emphasizing the large volumes of molecular data required to train algorithms, inflating privacy and data ownership challenges. The high demand for data might incentivize risky behavior, like suboptimal data handling and protection practices.

*“…to ensure anonymity*,* there must be a means to restrict access to this data to researchers*,* and to avoid that the data is sent anywhere” [INT 8]*

Eight participants agreed that while privacy is essential, restrictions should be balanced against the need to facilitate data access and sharing. Strict and heterogeneously implemented regulations (e.g., the European General Data Protection Regulation - GDPR) can hamper collaboration, research, and, ultimately, the broader implementation of ML in cancer care.

*“…access is very limited since we have a lot of ethical and regulatory issues that we need to solve” [INT 4]*

Six participants were less worried about privacy. If cancer patients provide informed consent for anonymized sharing, participants felt that privacy risks were minimal, especially for somatic tumor data. Some argued that privacy fears are overrated compared to the potential benefits of ML in oncology. They also emphasized that for most patients, what matters more than anything else is timely treatment without being hindered or delayed by overly strict privacy regulations.

*“You have a cancer*,* and you are metastatic. I don’t believe the patient has problem of privacy” [INT 6]*

### Focusing on equity

Participants argued for a greater focus on issues of equity and bias. One participant argued that as precision oncology becomes more sophisticated, those in greatest need are at risk of exclusion due to affordability, potentially widening inequity gaps within and among nations and socio-economic groups.

*“I believe that we should shift the ethical part*,* not so much to the protection of the data*,* that this is something that actually it should be with no doubt […] but is the research designed to offer solutions that will be affordable for most of us or we will need to be rich to have access to this?” [INT 7]*

Participants also mentioned that the concentration of data analytics skills in a few institutions with the required resources and infrastructure creates hierarchies and geographic access inequities.

*“…because it’s forcing a lot of the institutions to significantly increase the complexity of the diagnosis apparatus that they have*,* and that creates*,* if you want probably also*,* the need to have hierarchy” [INT 14]*

Molecular data is primarily collected in higher-income settings, leading to biased algorithms. One participant argued that some bias is inevitable and should not be the reason to reject the use of ML but rather a motivator to accelerate data collection for underrepresented populations.

*“…it is true that AI is as biased as the data sets that have been used to train the ML […] but I don’t think it’s a reason not to use ML […] it’s a reason to accelerate the generation of data that can be useful for […] underrepresented populations as fast as we can” [INT 1]*

Participants repeatedly mentioned the need to develop ML models that represent all people. Some suggested that algorithmic bias can also be mitigated through transparency and documenting how an algorithm is trained and tuned, including its performance in different demographics. ML developers need to transparently communicate for whom their algorithms will likely work best. In turn, healthcare professionals need to be aware of how an algorithm works across the spectrum of patients and use it accordingly.

*“And so the question of what are the ML algorithms being tuned on? And then how does that apply if you have a rare subtype. I don’t think it’s been very carefully looked at” [INT 2]*

Finally, molecular data needed to train and develop ML algorithms were described by one participant as the “people’s data”, implying that ML algorithms should be developed to benefit society as a whole.

### Explainability and trust

Explainability and trust were often discussed together. Participants argued that for trust to be built among patients and physicians, ML algorithms should provide transparency on the general logic of the algorithm, on how a certain clinical decision was reached, or how a given prediction is justified. Explainability was described as necessary in the context of clinical decision-making, as conflicts between physicians and algorithms can arise. This was described as particularly important for physicians with lower digital skill levels, who tend to trust and adopt technology less. Liability was also mentioned in case of damage incurred by errors related to ML decisions. Due to a lack of clinical integration, some participants described explainability and liability as future concerns.

*“AI should do all these jobs. And finally*,* should be also explainable*,* and this is a trend: Explainable AI. Because in order to adopt something*,* you need to understand” [INT 7]*

*“…but as soon as you go outside of the clinical intuition*,* and it’s really out-of-the-box prediction*,* [it is] going to be very difficult if you have a blackbox algorithm to convince the physician” [INT 11]*

While this is desired, participants acknowledged that explainability is a difficult concept, even more so when complex molecular data are involved. Two participants argued that explainability is overrated and not achievable without trading off the performance of an algorithm.

*“Yeah*,* well*,* you have always this trade-off between*,* I would say explainability and just performance […] we’re not going to have interpretable machine learning*,* and we’re just going to lose in performance if we do that” [INT 11]*

Beyond making ML outputs explainable, one participant also described the difficulty in translating “human language” into “machine-coherent language,” as the datasets we generate to train algorithms (referred to by our participant as “human language”) are prone to inconsistencies and gaps, and often not complete or coherent enough to adequately train ML.

*“But the reality is that it remains very difficult to translate human language into machine language” [INT 14]*

### Hype

Participants described several factors that can fuel problematic hype around ML in precision oncology. First, AI, specifically ML, can be portrayed as the ultimate solution to any medical condition.

*“…I think that there’s a miscommunication. Because I mean*,* AI is presented like the ultimate tool that will be used and will solve all of the problems” [INT 4]*

Second, there has been an over-emphasis on cancer cure. The promise that precision oncology and ML can dramatically improve clinical outcomes cannot be fulfilled yet. Yet, it is often communicated as such to secure research funding. Third, the hype is further fueled by vague terminologies, such as the term “precision.”

“…you think that genomic oncology will cure you. And that’s not the case yet. And I think that’s a big downside. We overpromised in comparison to what can be delivered today.” [INT 1]

Participants also mentioned negative hype, or the fear and overemphasized risks of ML. All of this leads to polarized views between those who see ML as a magic bullet and those who primarily see the risks.

“…we are a little bit very fans of making movies about all the bad things that could happen, even though at the same time, there are many good things that will happen for sure if we use the methods” [INT 1]

One participant suggested that instead of promoting ML as a revolution, generating a divisive bi-directional hype, it should be promoted as an evolution that will not outpace but improve medicine as we know it.

*“And I think the best way forward would be to say that it just yet another tool[…]*,* and nothing revolutionary. It’s an evolution; it’s not a revolution. And it’s an evolution that*,* in many cases*,* will be very helpful.” [INT 1]*

### Future outlook

Overall, almost all participants agreed that ML is not just a temporary hype but the future of precision oncology, most likely in an assistive rather than decision-making role. Participants expect ML to homogenize decision-making and accelerate drug discovery in the long term. Some described that while future ML tools might provide personalized cancer care recommendations, the decision-making power of oncologists will remain, as factors such as personal circumstances and patient preferences can only be captured by physicians.

*“I don’t see this as just like a kind of hype that is going to be down by five or ten years of now. […] I think it’s taken off*,* and it’s not going to finish in a few years.” [INT 11]*

*“I mean*,* of course*,* I think that it will accelerate drug discovery […]*,* so it will be sustainable and affordable” [INT 3]*

*“…of course*,* you can […] probably create a ranking based on the data […]*,* but I mean*,* I think you always require human experts that actually look at those data and*,* and sort of also figure it in the personal aspects of the patient”* [INT 10]

Three participants underlined ML’s role in discovering new biomarkers that can predict cancer outcomes and thus facilitate better, more affordable, and sustainable care. ML becomes particularly relevant for analyzing and understanding the increasingly important role of multiple molecular alterations in personalized cancer care.

*“Well*,* obviously*,* we cannot find the predictive variables that are going to be used in the future without machine learning. There is no way because there are so many variables to work with” [INT8]*

Five participants mentioned the need for closer dialogue and collaboration between oncologists, computer scientists, and bioethicists to ensure that research fulfills actual clinical needs and ethical standards. Participants believed that having stakeholders disconnected from one another would ultimately hinder the clinical prospects of using ML in precision oncology.

*“One of the things critical for me in any project it is always having a tandem between the scientist and a clinician because you can try to come up with answers*,* but if it’s something that is not a question for physicians*,* you’re wasting your time.” [INT 9]*

Finally, six participants mentioned the need for more awareness and capacity building across all stakeholders. This includes educating physicians on the limitations of ML, their responsibilities when working with ML, and how to approach conflicts between ML and the physicians’ clinical judgment. Participants also emphasized the need to sensitize patients and citizens on precision oncology, genomics, and the critical role of ML. One participant highlighted the need to help patients understand and engage with personal risk and the related uncertainties.

*“…right now*,* I don’t think clinicians have the background and to really grasp all the implications of those new tools of machine learning*,* but I hope this is going to change” [INT 11]*

*“I think more people should be educated to assessing engaging their personal risk so that they need to grasp probability theory in a certain sense” [INT 14]*

## Discussion

Our participants recognized the potential of ML for future cancer care, highlighting its ability to address the dynamic nature of cancer, break down the high complexity of molecular data, and support decision-making. Yet, they emphasized several challenges. Some of our participants saw the availability of reliable data sources as critical for the clinical implementation of ML, echoing similar considerations frequent in AI-driven biomedicine literature [17, 18]. Impediments to data availability include the lack of data-sharing incentives, insufficient digitalization of phenotypic and clinical information, under-sampled patient groups, consent restrictions, privacy concerns, and stringent data protection requirements [19–23]. Others argued that the clinical utility of ML will be predicated upon efficient integration of multi-omic data into other clinical data [6]. Our participants noted this remains an unfulfilled potential, highlighting an important translational gap [24].

Our data also supported other well-known challenges to the clinical application of ML, such as algorithmic bias [25–27]. Using biased datasets in ML training leads to differential performance for different patient groups. Yet large initiatives, such as TCGA or the Pan-Cancer Analysis of Whole Genomes, primarily include data from people of European ancestry [28, 29]. In particular, socio-demographic characteristics like race, ethnicity, gender, and income should be carefully controlled to limit the likelihood of bias [30]. Our participants acknowledged the risk of bias and argued for training datasets that are truly representative of the target population. Representative training datasets are recognized as the main driver of fair and equitable ML. However, biases can emerge even downstream of residual biases and should be tested for and documented, arguably requiring specific knowledge and skills on the part of physicians to ensure fair clinical outcomes.

Explainability is another concern in medical AI, with regulators pushing for at least some degree of transparency regarding the general logic or the individual decisions, predictions, and classifications offered by AI algorithms in medicine and beyond [31, 32]. In our data, explainability emerged as an essential determinant of clinical uptake. However, in line with recent reports in the literature highlighting the technical challenges of explainable ML, some participants also questioned the centrality of explainability vis-á-vis clinical utility as the pivotal element driving clinical implementation [33].

Opinions on ethical concerns varied, with privacy being the most polarizing aspect. Some participants were highly concerned, and others were less concerned. European participants often cited strict data protection laws as a barrier to accessing molecular data, highlighting the need for a better balance between privacy and data sharing for scientific progress. Others called for a shift in focus from privacy to equity, suggesting that privacy discussion has become oversaturated [27]. Overall, discussions about the ethical and regulatory aspects of digital health and big data-driven health research primarily revolved around privacy, data protection, and equity [34–36].

Our study participants emphasized concerns linked to data availability and data protection. In this sense, our results revealed participants’ awareness of ML’s ethical implications. However, the study did not pinpoint specific issues regarding clinical decision-making, reflecting an underappreciation of this topic in the literature. Notably absent from the literature are considerations on how to communicate ML-generated predictive information to patients and how molecular tumor boards should handle predictions about potentially effective molecules. For instance, an ML algorithm may predict that a given compound currently under investigation may benefit a given patient. The oncologist could then invite that patient to join a clinical trial or file an expanded access (compassionate use) request. If AI predicts benefit from a drug approved for a different oncologic indication, a provider could prescribe the molecule off-label under her clinical supervision. To the extent that ML models are more likely to be used for patients who do not respond to standard treatment or for late-stage cancers in general, clear guidelines are needed to help tumor boards orient themselves, configuring what could be termed “experimental care.” Failure to standardize clinical practices may hinder the clinical integration of ML models. Similarly, special attention should be paid to the risk of therapeutic misconception on the part of trial participants, that is, the risk that oncology patients enroll in trials under the false expectation it is designed to meet their individual health needs [37].

While ML models can predict the most effective targeted therapy for a given patient, clinical judgment and patient involvement are still needed to assess risks (e.g., toxicity) and benefits in light of individual patient preferences [38]. Specific training is therefore needed to enable healthcare professionals to clinically handle ML-driven predictions and integrate them into existing clinical workflows.

Similarly, ethics committees should have a sufficient understanding of medical ML in oncology to provide guidance and to effectively oversee its clinical use.

Research efforts in the field of AI-driven precision oncology are well underway. Recently released large language models offering user-friendlier ways to analyze individual patient cases hold promise to dramatically accelerate the development of ML for oncological use as well as its clinical implementation– arguably posing a host of specific ethical challenges that remain to be explored [39]. Such developments offer enhanced opportunities for AI integration as precision oncology itself develops to embrace functional approaches to precision medicine aimed at integrating genomic analysis with testing drugs ex vivo directly on each individual patient cancer cells [40, 41].

As the field rapidly expands, professional and regulatory guidance is needed to fill gaps in data protection, algorithmic fairness, and explainability regarding optimal clinical implementation, liability, and reimbursement.

Data access and sharing, as also highlighted by our participants, represent limiting factors for the development of AI-guided precision oncology and its clinical implementation. In this context, international initiatives like the FAIR Data Principles have stressed the importance of promoting machine-driven automatic finding and use of scholarly data [42]. To support the use of AI in precision oncology, FAIR Data Principles should arguably expand to the clinical setting to enable AI models to improve as they are used.

## Conclusions

Our work offers insight into experts’ perspectives on ML models’ challenges, opportunities, and outlook for precision oncology. Given the unique nature of medical AI, our findings highlight the field’s potential and remaining challenges [43]. ML will continue to advance cancer research and provide opportunities for patient-centric, personalized, and efficient precision oncology. Yet, the field must move beyond hype and toward concrete efforts to overcome key obstacles, such as ensuring access to molecular data, establishing clinical utility, developing guidelines and regulations, and meaningfully addressing ethical challenges. Such efforts will be key to demonstrating clinical value.

## Electronic supplementary material

Below is the link to the electronic supplementary material.

Supplementary Material 1

## Data Availability

The datasets used and/or analyzed during the current study available from the corresponding author on reasonable request.
