# Supplementary material for: Realizing the promise of machine learning in precision oncology: expert perspectives on opportunities and challenges
Source: BMC Cancer. 2025 Feb 17;25:276. doi: 10.1186/s12885-025-13621-2 (PMC11834663; doi:10.1186/s12885-025-13621-2)
Supplement: Supplementary file 1 — Supplementary Material 1 [file 12885_2025_13621_MOESM1_ESM.pdf]

© ETH Zurich – V. Nittas, A. Blasimme

Interview Guide

PART 1

1. How familiar are you with the notion of precision oncology and how would you define it?
2. What do you think about the potential impact of precision oncology on patient care and outcomes?
3. What role do genetic and/or genomics data should have in clinical decision making in oncology?
4. What role should genetic and/or genomics data should have in clinical decision-making in oncology? Where do you see its strengths/benefits?
5. Do you envisage any specific downsides of implementing of precision oncology (using genetics and genomics for decision making)?
6. [if not covered in the answer to the previous one] Do you envisage any specific ethical challenges of precision oncology (using genetics and genomics for decision making)?
7. Are you familiar with / are you a member of / have you ever interacted with a molecular tumor board? If so, what impact do you think MTBs have on patient care and outcomes?

PART 2

1. AI/ML can advance the analysis and interpretation of genomic data. How familiar are you/ what is your experience with using AI/ML in precision oncology?
2. What role do you think AI/ML could have in precision oncology? Where do you see its strengths/benefits?
3. In your opinion,what hurdles the use of AI/Ml in precision oncology?
4. Do you envisage any specific downsides of implementing AI in precision oncology?
   - Do you see any differences from other clinical settings?
5. [if not covered in the answer to the previous one]Do you envisage any specific ethical challenges of implementing Ai in precision oncology
   - Do you see any differences from other clinical settings?
